# Supplementary material for: Comparative Roles of Rad4A and Rad4B in Photoprotection of Beauveria bassiana from Solar Ultraviolet Damage
Source: J Fungi (Basel). 2023 Jan 23;9(2):154. doi: 10.3390/jof9020154 (PMC9961694; doi:10.3390/jof9020154)
Supplement: Supplementary file 1 [file jof-09-00154-s001.zip › jof-2156704-supplementary.pdf]

Supplementary Material for Journal of Fungi

Comparative Roles of Rad4A and Rad4B in Photoprotection of *Beauveria bassiana* from Solar Ultraviolet Damage

Lei Yu, Si-Yuan Xu, Xin-cheng Luo, Sheng-Hua Ying, Ming-Guang Feng *

Institute of Microbiology, College of Life Sciences, Zhejiang University, Hangzhou 310058, China

* Correspondence: mgfeng@zju.edu.cn; Tel/Fax: (+)86-571-8820-6178

Table S1. Paired primers used for manipulation and detection of *rad4A* and *rad4B* in *B. bassiana* and Y2H assays for protein-protein interactions.

Primers	Paired sequences (5′–3′)*	Purpose
cRad4A-F/R	CAATCACAAACACCTTCAAAATGGTCGGAAGAAAGCGGCG / TCCTCGCCCTTGCTCACCATATCTACAAGAAATCCACCAC	Cloning *rad4A* cDNA (2517 bp) for fusion to *gfp*
cRad4B-F/R	CAATCACAAACACCTTCAAAATGCCACCACATGTACCGCG / TCCTCGCCCTTGCTCACCATTGTCTCATCCTCCAAGACCA	Cloning *rad4B* cDNA (2721 bp) for fusion to *gfp*
cRad23-F/R	CAATCACAAACACCTTCAAAATGAAGGTCACCTTCAGAGA / TCCTCGCCCTTGCTCACCATTTGGCTCGCAGGCGGCTGCT	Cloning *rad23* cDNA (1191 bp) for fusion to *mCherry*
adRad4A -F/R	GCCATGGAGGCCAGTGAATTCATGGTCGGAAGAAAGCGGCG / CAGCTCGAGCTCGATGGATCCATCTACAAGAAATCCACCAC	Cloning *rad4A* cDNA (2517 bp) for ligation to AD
bdRad4A -F/R	ATGGCCATGGAGGCCGAATTCATGGTCGGAAGAAAGCGGCG / CGCTGCAGGTCGACGGATCCATCTACAAGAAATCCACCAC	Cloning *rad4A* cDNA (2517 bp) for ligation to BD
adRad4B -F/R	GCCATGGAGGCCAGTGAATTCATGCCACCACATGTACCGCG / CAGCTCGAGCTCGATGGATCCTGTCTCATCCTCCAAGACCA	Cloning *rad4B* cDNA (2721 bp) for ligation to AD
bdRad4B -F/R	ATGGCCATGGAGGCCGAATTCATGCCACCACATGTACCGCG / CGCTGCAGGTCGACGGATCCTGTCTCATCCTCCAAGACCA	Cloning *rad4B* cDNA (2721 bp) for ligation to BD
adRad23 -F/R	GCCATGGAGGCCAGTGAATTCATGAAGGTCACCTTCAGAGA / CAGCTCGAGCTCGATGGATCCTTGGCTCGCAGGCGGCTGCT	Cloning *rad23* cDNA (1191 bp) for ligation to AD
bdRad23 -F/R	ATGGCCATGGAGGCCGAATTCATGAAGGTCACCTTCAGAGA / CGCTGCAGGTCGACGGATCCTTGGCTCGCAGGCGGCTGCT	Cloning *rad23* cDNA (1191 bp) for ligation to BD
adWC1-F/R	GCCATGGAGGCCAGTGAATTCATGGAAGGATACTACCCTCC / CAGCTCGAGCTCGATGGATCCAGGTAAGCTCGTTTCACGCT	Cloning *wc1* cDNA (2889 bp) for ligation to AD
adWC2-F/R	GCCATGGAGGCCAGTGAATTCATGTCCCAGGGACACGCGCC / CAGCTCGAGCTCGATGGATCCGCTTCCGGCACCAAACTCTG	Cloning *wc2* cDNA (1497 bp) for ligation to AD)
adPhr1-F/R	GCCATGGAGGCCAGTGAATTCATGGCGCCTCGTGCAACGAA / CAGCTCGAGCTCGATGGATCC CGCCACAGCCGCCTTGTACG	Cloning *phr1* cDNA (1761 bp) for ligation to AD
bdPhr1-F/R	ATGGCCATGGAGGCCGAATTCATGGCGCCTCGTGCAACGAA / CGCTGCAGGTCGACGGATCCCGCCACAGCCGCCTTGTACG	Cloning *phr1* cDNA (1761 bp) for ligation to BD
adPhr2-F/R	GCCATGGAGGCCAGTGAATTCATGACAAAGCCCAGAGTCAT / CAGCTCGAGCTCGATGGATCCCGTTTTCTGCTTCTTCGCTG	Cloning *phr2* cDNA (1869 bp) for ligation to AD
bdPhr2-F/R	ATGGCCATGGAGGCCGAATTCATGACAAAGCCCAGAGTCAT / CGCTGCAGGTCGACGGATCCCGTTTTCTGCTTCTTCGCTG	Cloning *phr2* cDNA (1869 bp) for ligation to BD
upRad4A -F/R	ACGAGCTGTACAAGTAACCCGGGGACCCAGTTCTGCTCCAT / TGGCTGCAGGTCGACGGATCCCGCCAAAGTGCGTCATAG	Cloning *rad4A* 5'-end (1787 bp) for disruption
dnRad4A -F/R	GACCCATGGCTCGAGTCTAGACTCAAGGGCATCGTGGTC / GGTGGTGGTGGCTAGCGTTAACGAGGCAAACGGAGGGGAC	Cloning *rad4A* 3'-end (1844 bp) for disruption
upRad4B -F/R	ACGAGCTGTACAAGTAACCCGGGATCTCATCGAGGTCTAGCA / TGGCTGCAGGTCGACGGATCCTGTAATCGCCTGTTCACG	Cloning *rad4B* 5'-end (1871 bp) for disruption
dnRad4B -F/R	GACCCATGGCTCGAGTCTAGAGAGCCTACCACTAACCACAGC / GGTGGTGGTGGCTAGCGTTAACATGGAGCCGAGACCGATT	Cloning *rad4B* 3'-end (1778 bp) for gene disruption
flRad4A -F/R	ATCCGTCGACCTGCAGCCAAGCTTACTGATAATCCGTGACTGGTG /ACACTAGTCAGATCTTCTCTAGACGGTTCCCGAATAGAGCA	Cloning full-length *rad4A* (6107 bp) for complementation
flRad4B -F/R	ATCCGTCGACCTGCAGCCAAGCTTGGGGAAGTGCCAAGAAGG / ACACTAGTCAGATCTTCTCTAGAATGGAGCCGAGACCGATT	Cloning full-length *rad4B* (6100 bp) for complementation
pRad4A -F/R	GCTTTCCTTTTCGTTGTCAG / CCTGCTCTTGTTCCACATTCT	PCR detecting *rad4A*
pRad4B -F/R	TTATTTACCCCATCTGACTGCT / TTCGGCAACTACGACACCT	PCR detecting *rad4B*

* The underlined regions are DNA fragments to exchange for the corresponding fragments of constructed vectors at the sites (double-underlined) of restriction enzymes (*Eco*RI/*Bam*HI for ligation to AD or BD, *Xma*I/*Bam*HI and *Xba*I/*Hpa*I for deletion vectors of *rad4A* and *rad4B*, and *Hin*dIII/*Xba*I for complementation vectors of *rad4A* and *rad4B*).

Table S2. Paired primers used for transcriptional analysis of anti-UV genes in *B. bassiana*.

Gene	Tag locus*	Annotation	Sequences (5′–3′) of paired primers
rad4A	BBA_02814	Rad4 homolog 1	AGTCCGATATGCAAAGGCGT / TACTTCGTGGCGCCGTAAAT
rad4B	BBA_02963	Rad4 homolog 2	CCTGATGGTACAGCCAAGGA/CGTGCTCTCTTCCACTTCGT
wc1	BBA_10271	White collar 1	GACCATGCAATTCAACAACG / GTATCGCTTGATCGACAGCA
wc2	BBA_01403	White collar 2	CCAGTCTCCTTTCTGCCAAG / TGGCAGATGAACCAGTCAAG
phr1	BBA_01664	CPD photolyase	ACTCATAGACTGGCGCATGG / TTTTCGCCTTGTCTCCAGCA
phr2	BBA_01034	6-4 PP photolyase	CACAGGCAAGACGTACCCC / CGTCGTCACTCTCCAGAACA
cryD	BBA_02424	DASH-type cryptochrome	CGTATTACCTCCCGACCAGA/GCACTGCAGATGCTGGATAA
rad23	BBA_01030	Rad23 ortholog	AGCAGAAATTCACCCTCGAA / CAGACAACGAAGCCCTTTTC
act	BBA_04860	β-actin	GGCAACATTGTCATGTCTGG / TTTGCTGGAAGGTGGATAGG

* Gene accession codes in *B. bassiana* genome under the NCBI accession NL_ADAH00000000.

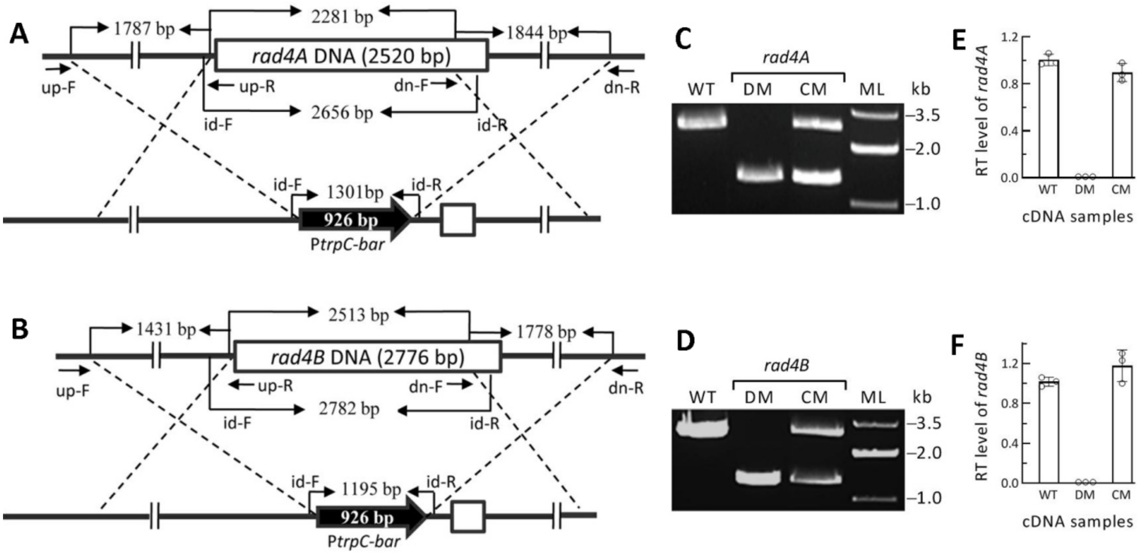

Figure S1. Generation and identification of *rad4A* and *rad4B* mutants in *B. bassiana*. (**A, B**) Schematic diagrams for targeted single-gene deletion strategies. (**C, D**) The *rad1, rad10, wc1* and *wc2* mutants identified via PCR analysis with paired primers (see Table S1), respectively. The detected DNA fragments indicate a success in deleting the partial or full-length coding and partial flanking regions of each target gene from the WT strain as expected and also in complementing it into the deletion mutant (2782 + 926 – 1301 = 2281 bp for *rad4A*, and 2782 + 926 – 1195 = 2513 bp for *rad4B*). DM, deletion mutant. CM, complementation mutant. ML, molecular ladder of genomic DNA. (**E, F**) Relative transcript (RT) levels of *rad4A* and *rad4B* in the 3 d-old SDAY cultures of their DM and CM strains with respect to the WT standard. Note that the expression of each target gene was not detectable in its DM but well restored in its CM. Error bars: standard deviations of the means from three cDNA samples derived from independent cultures of each strain.

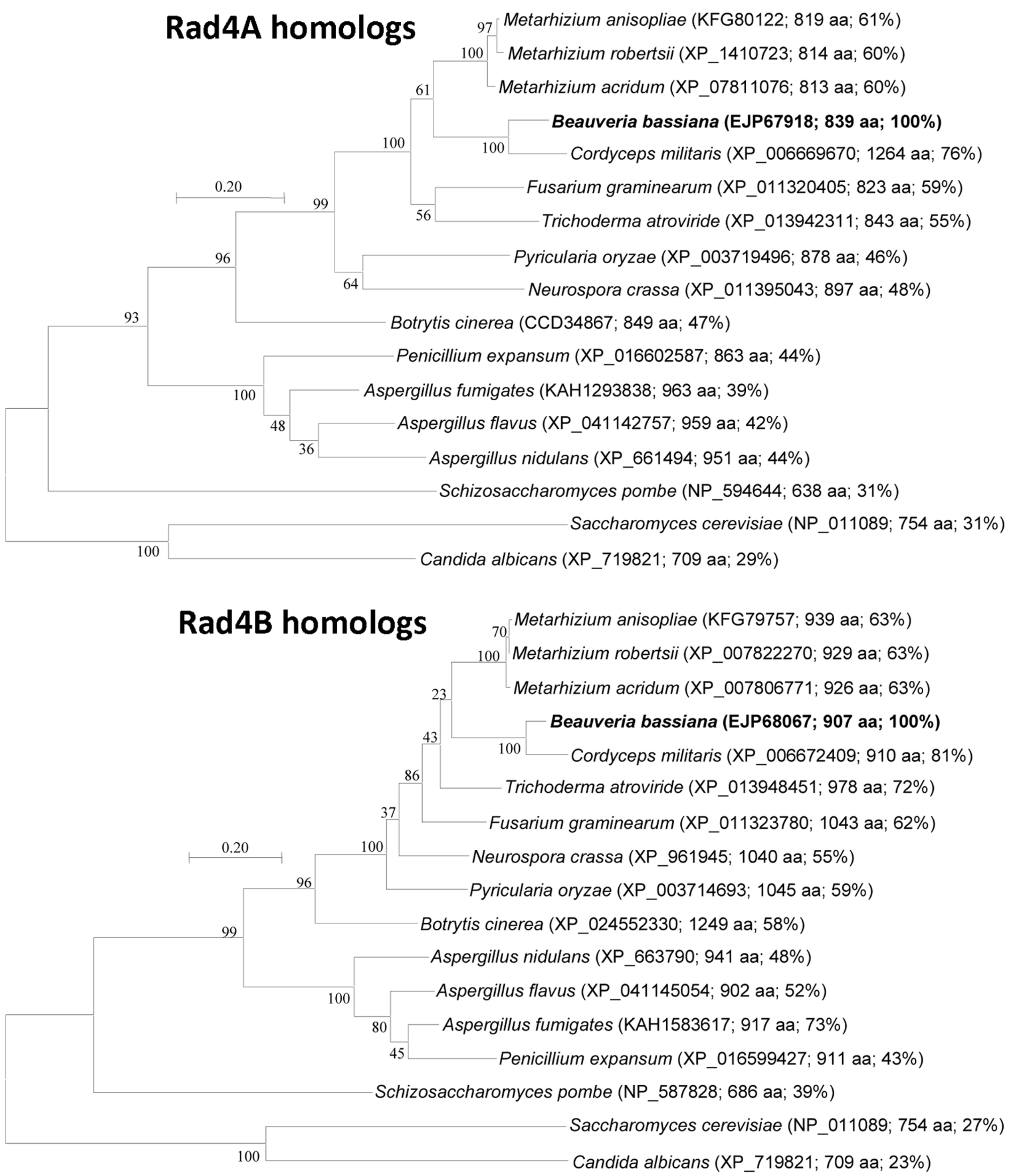

Figure S2. Phylogenetic trees of Rad4A and Rad4B homologs found in representative ascomycetes. Each tree was constructed with the maximum likelihood method in MEGA11 (http://www.megasoftware.net/). Bootstrap values of 1000 replications are shown at nodes. Scale bar: branch length proportional to genetic distance. The NCBI accession code of each protein and its protein sequence identity to its *B. bassiana* homolog (in bold) are given in the parentheses following the name of its host fungus. Note that Rad4A and Rad4B paralogs exist in all surveyed fungal genomes except *S. cerevisiae* and *C. albicans*.

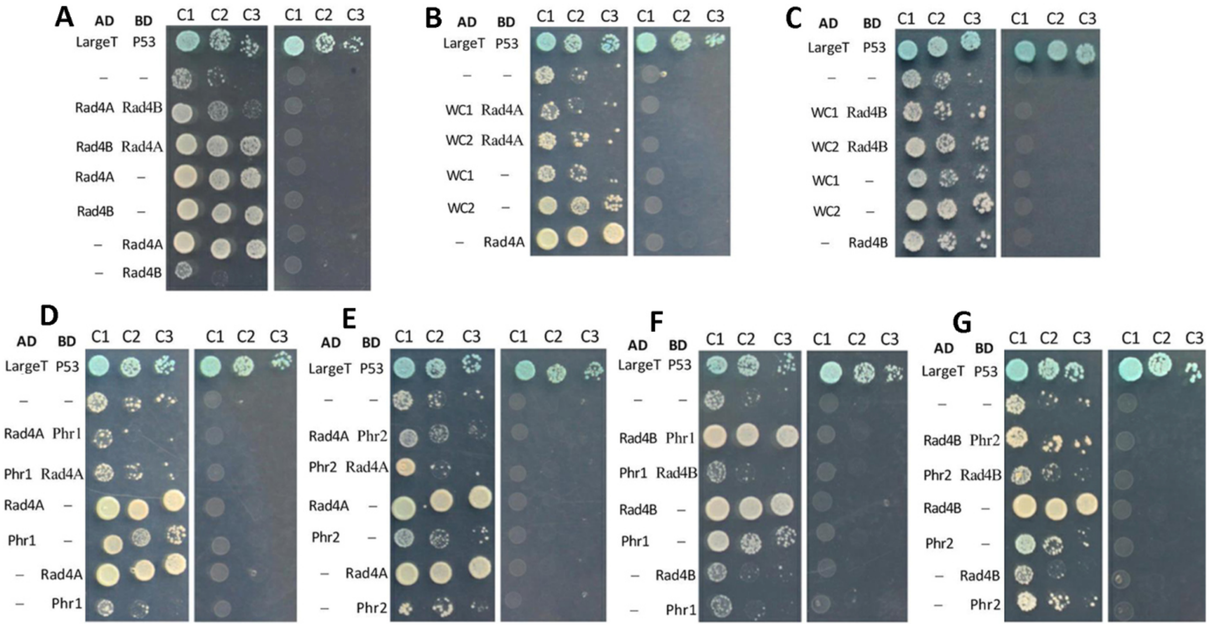

Figure S3. Y2H assays for protein-protein interactions in *B. bassiana*. Note no signals for interactions between Rad4A and Rad4B (**A**), between Rad4A and WC1 or WC2 (**B**), between Rad104B and WC1 orWC2 (**C**), between Rad4A and Phr1 (**D**) or Phr2 (**E**), and between Rad4B and Phr1 (**F**) or Phr2 (**G**).

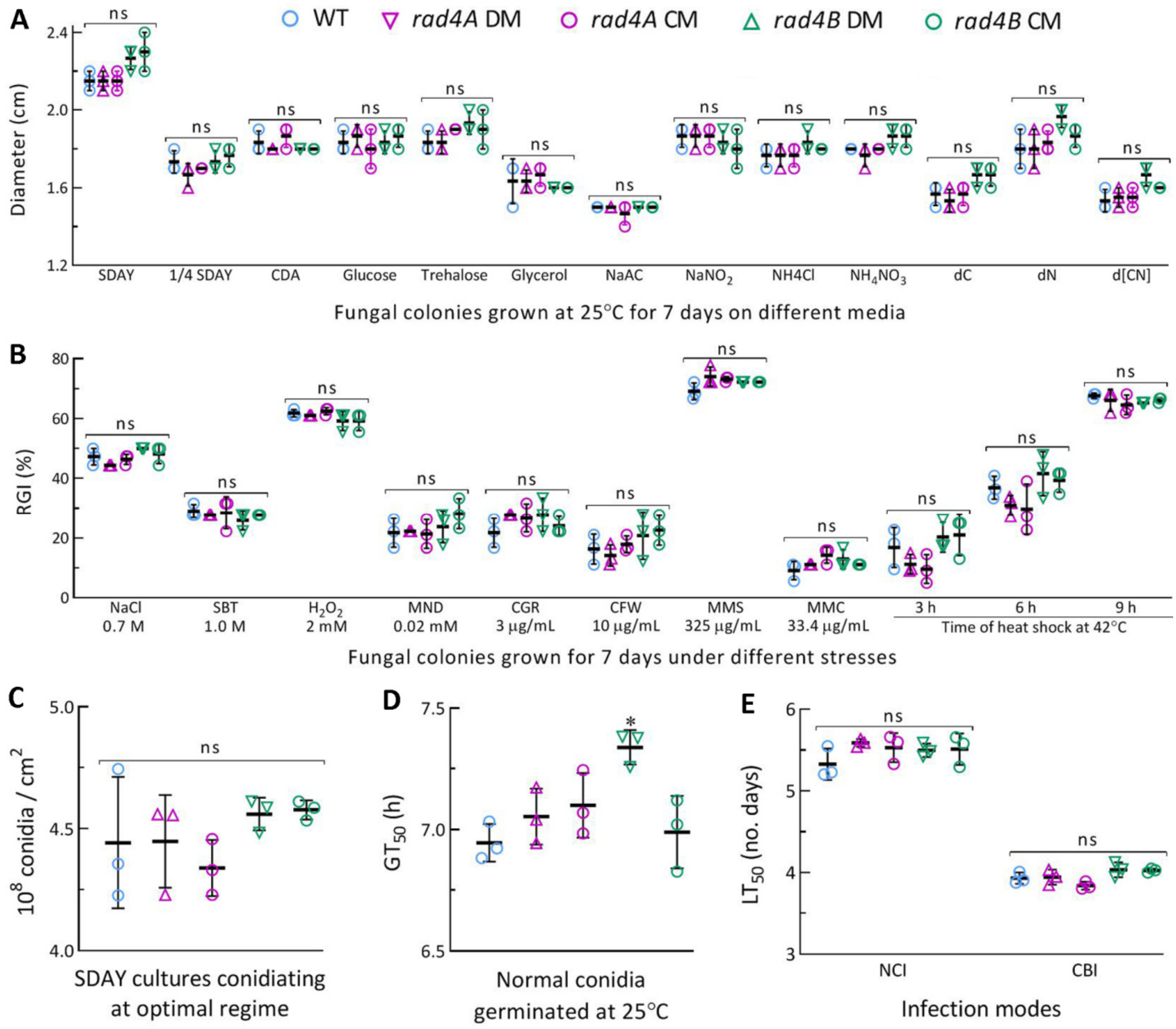

Figure S4. Rad4A and Rad4B are dispensable for the lifecycle in vitro and in vivo of *B. bassiana*. (**A**) Diameters of fungal colonies (DM, deletion mutant; CM, complementation mutant) incubated at 25°C for 7 days on the plates of rich medium SDAY, 1/4 SDAY, minimal medium CDA and CDAs amended with different carbon or nitrogen sources and with the deletion of carbon (d[C]) or nitrogen (d[N]) source or both (d[CN]). (**B**) Relative growth inhibition (RGI) percentages of fungal colonies incubated at 25°C for 7 days on CDA plates supplemented with indicated concentrations of different chemical stressors (SBT, sorbitol; MND, menadione; CGR, Congo red; CFW, calcofluor white; MMS, methyl methanesulfonate; and MMC, mitomycin C) or for 5-day growth recovery after exposing 2-day-old SDAY colonies to a heat shock for 3, 6 and 9 h at 42°C. All fungal colonies were initiated by spotting 1 µL aliquots of a 10^6 conidia/mL suspension. (**C**) Conidial yields measured from the 7-day-old SDAY cultures initiated by spreading 100 µL aliquots of a 10^7 conidia/mL suspension at the optimal regime of 25°C and L:D 12:12. (**D**) Median germination time (GT_{50}) of conidia at 25°C. (**E**) Median lethal time (LT_{50}) estimates for fungal strains against *Galleria mellonella* larvae (4th instar) inoculated by topical application (immersion) of a 10^7 conidia/mL for normal cuticle infection (NCI) or intrahemocoel injection ~500 conidia per larva for cuticle-bypassing infection (CBI). $P<0.05$* or >0.05 (ns, not significant) in Tukey's tests. Error bars: standard deviations of the means from three independent replicates.
